# Genome-wide association study reveals the genetic basis of fiber quality traits in upland cotton (*Gossypium hirsutum* L.)

**DOI:** 10.1186/s12870-020-02611-0

**Published:** 2020-08-27

**Authors:** Wei Liu, Chengxiang Song, Zhongying Ren, Zhiqiang Zhang, Xiaoyu Pei, Yangai Liu, Kunlun He, Fei Zhang, Junjie Zhao, Jie Zhang, Xingxing Wang, Daigang Yang, Wei Li

**Affiliations:** 1 Collaborative Innovation Center of Henan Grain Crops, Agronomy College, Henan Agricultural University, Zhengzhou, 450002 China; 2 State Key Laboratory of Cotton Biology, Institute of Cotton Research, Chinese Academy of Agricultural Sciences, Anyang, 455000 China; 3 Zhengzhou Research Base, State Key Laboratory of Cotton Biology, Zhengzhou University, Zhengzhou, 450001 China

**Keywords:** Upland cotton, Fiber quality, Genome-wide association study, Single nucleotide polymorphism, Quantitative trait locus

## Abstract

**Background:**

Fiber quality is an important economic trait of cotton, and its improvement is a major goal of cotton breeding. To better understand the genetic mechanisms responsible for fiber quality traits, we conducted a genome-wide association study to identify and mine fiber-quality-related quantitative trait loci (QTLs) and genes.

**Results:**

In total, 42 single nucleotide polymorphisms (SNPs) and 31 QTLs were identified as being significantly associated with five fiber quality traits. Twenty-five QTLs were identified in previous studies, and six novel QTLs were firstly identified in this study. In the QTL regions, 822 genes were identified and divided into four clusters based on their expression profiles. We also identified two pleiotropic SNPs. The SNP locus i52359Gb was associated with fiber elongation, strength, length and uniformity, while i11316Gh was associated with fiber strength and length. Moreover, these two SNPs were nonsynonymous and located in genes *Gh_D09G2376* and *Gh_D06G1908*, respectively. RT-qPCR analysis revealed that these two genes were preferentially expressed at one or more stages of cotton fiber development, which was consistent with the RNA-seq data. Thus, *Gh_D09G2376* and *Gh_D06G1908* may be involved in fiber developmental processes.

**Conclusions:**

The findings of this study provide insights into the genetic bases of fiber quality traits, and the identified QTLs or genes may be applicable in cotton breeding to improve fiber quality.

## Background

Cotton (*Gossypium* spp.) is an important economic crop, and the major source of natural fibers for the textile industry worldwide [1]. Upland cotton (*Gossypium hirsutum* L.), an allotetraploid species, has the advantages of wide adaptability and high production. Consequently, it is used to produce approximately 95% of the world’s cotton fiber [2]. In the past few decades, cotton breeders have been mainly focused on improving cotton yield. At present, with improvements in the quality of consumers’ lives and in textile technology, the demand for high-quality fiber is increasing [3, 4]. Thus, improving fiber quality has become a new target of cotton breeding. However, improving the fiber quality of upland cotton by conventional breeding has proven to be inefficient [5]. Therefore, it is important to clarify the genetic bases of upland cotton fiber quality traits, and molecular markers will play vital roles in the breeding of high-quality cotton.

The fiber quality traits of cotton mainly include fiber elongation (FE), fiber micronaire (FM), fiber strength (FS), fiber length (FL) and fiber uniformity (FU). These are all complex quantitative traits that are substantially affected by environmental factors, even though they are mainly controlled by genetic factors [5]. Before cotton genomes were published, cotton fiber quality traits were mainly studied using linkage analysis methods with biparental segregating populations. To date, nearly 1000 quantitative trait loci (QTLs) associated with fiber quality traits and distributed across the 26 cotton chromosomes have been reported using this mapping method [6, 7]. These studies provided important information for fiber-related genetics and accelerated the development of cotton breeding for fiber quality.

In recent years, with the release of cotton genome sequences [8–10] and the rapid development of molecular markers [11, 12], a large number of single nucleotide polymorphism (SNP) markers have been identified at the whole-genome level in cotton. Additionally, association mapping has been widely used to discover the genetic bases of complex traits in cotton [13–16]. Compared with traditional linkage mapping, association mapping can reveal the associations between genotypes and phenotypes using natural populations and simultaneously detect many natural allelic variations in a study [17]. Using this mapping method, many QTLs and candidate genes associated with fiber quality have been identified [18]. For example, Sun et al. (2017) performed a genome-wide association study (GWAS) of fiber quality traits using 719 diverse accessions of upland cotton and 10,511 polymorphic SNPs identified using the CottonSNP63K array. In total, they identified 46 significant SNPs associated with five fiber quality traits. Furthermore, a combined GWAS and transcriptome analysis revealed 19 promising genes related to FL and FS [19]. Huang et al. (2017) detected 79 significant SNPs associated with fiber quality traits using a natural population containing 503 *G. hirsutum* accessions through CottonSNP63K genotyping [20]. Moreover, the genes *GhXI-K*, *GhFL1* and *GhFL2* were also identified as being associated with fiber quality traits using the association mapping method [13, 21].

In our previous study, 276 upland cotton accessions were genotyped with the CottonSNP63K array and 10,660 high-quality SNPs were identified [22]. Here, we performed a GWAS to determine the correlations between genetic loci and fiber quality traits using these 276 diverse accessions and 10,660 high-quality SNPs. Moreover, we also mined for candidate genes underlying the fiber quality traits and assessed them by integrating RNA-seq and RT-qPCR analyses. Our results are useful for the breeding of improved fiber quality traits in cotton.

## Results

### Phenotypic characterization of fiber quality traits

**Fig. 1:**
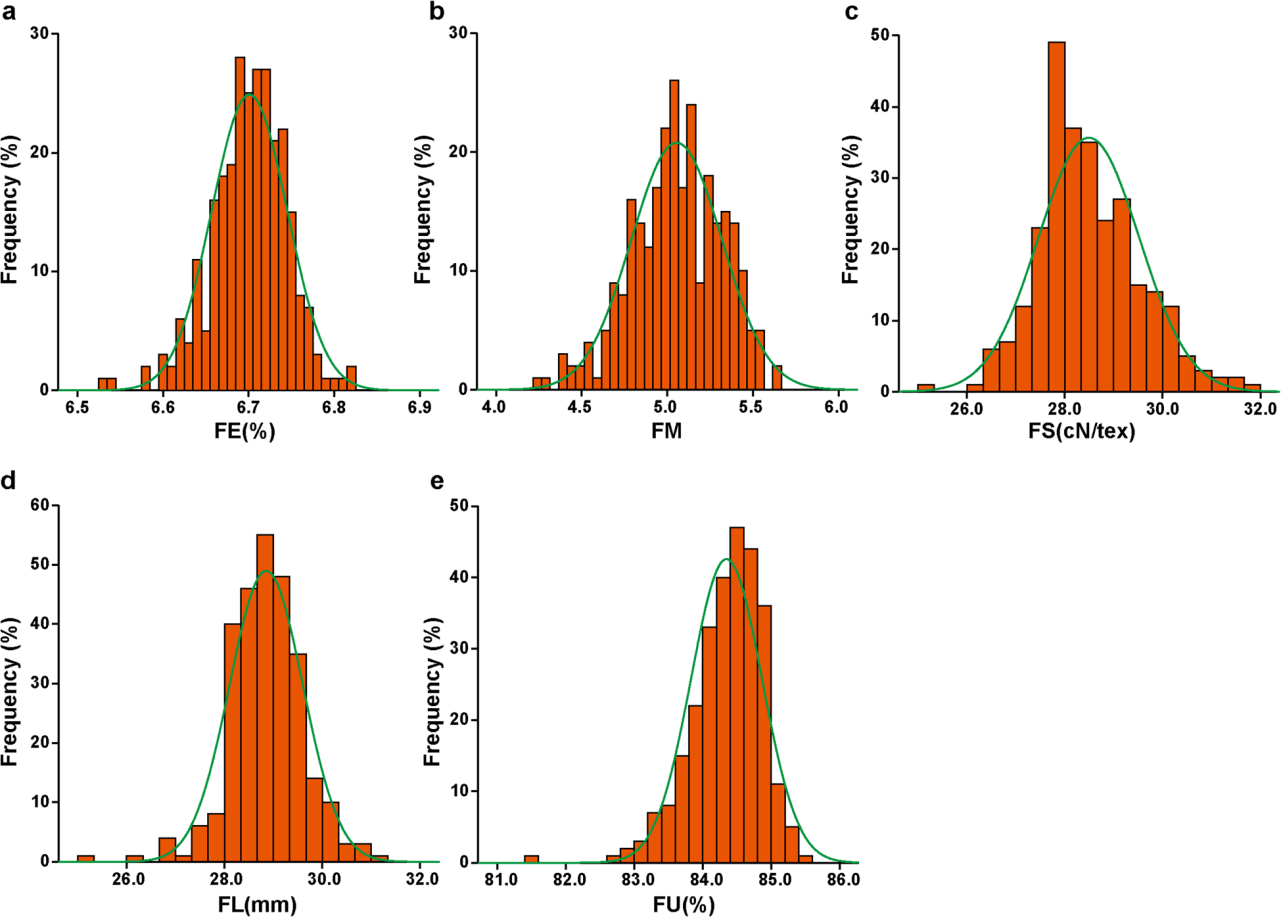
Frequency distributions of five fiber quality traits in the natural upland cotton population. **a** fiber elongation (FE), **b** fiber micronaire (FM), **c** fiber strength (FS), **d** fiber length (FL) and **e** fiber uniformity (FU)

The 276 diverse upland cotton accessions were obtained from different ecological zones, and the best linear unbiased predictions (BLUPs) of the phenotypic data exhibited abundant variation among accessions. FE, FM, FS, FL and FU exhibited values in the ranges of 6.53%–6.82%, 4.24–5.63, 25.08–31.69 cN/tex, 25.17–31.02 mm and 81.55%–85.41%, with average values of 6.70%, 5.05, 28.49 cN/tex, 28.85 mm and 84.35%, respectively (Table 1). All the fiber quality traits were normally distributed (Fig. 1), indicating that these traits were quantitative traits controlled by multiple genes. The coefficients of variation (CVs) of FE, FM, FS, FL and FU were 0.66%, 5.24%, 3.61%, 2.60% and 0.61%, respectively (Table 1).

**Table 1:** Phenotype statistics of fiber quality traits in the accession population

Trait	Min	Max	Mean	SD	CV(%)	*H*^***2***^(%)
FE(%)	6.53	6.82	6.70	0.04	0.66	73.31
FM	4.24	5.63	5.05	0.26	5.24	91.38
FS(cN/tex)	25.08	31.69	28.49	1.03	3.61	84.78
FL(mm)	25.17	31.02	28.85	0.75	2.60	84.54
FU(%)	81.55	85.41	84.35	0.52	0.61	72.06

*FE* Fiber elongation, *FM* Fiber micronaire, *FS* Fiber strength, *FL* Fiber length, *FU* Fiber uniformity, *SD* Standard deviation, *CV* Coefficient of variation, *H*^*2*^ Broad-sense heritability

**Fig. 2:**
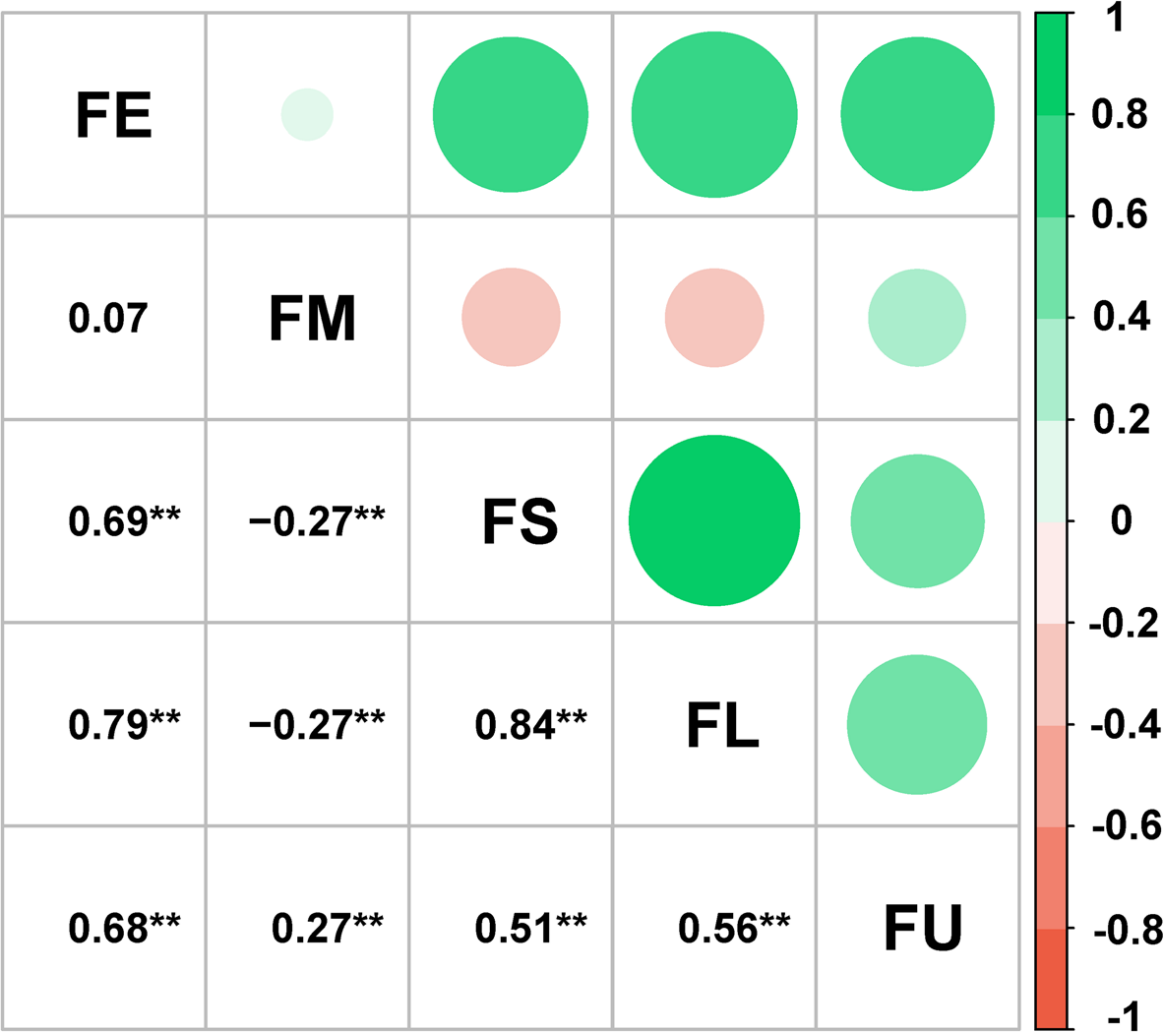
Correlation analysis of five cotton fiber quality traits. FE, fiber elongation; FM, fiber micronaire; FS, fiber strength; FL, fiber length; FU, fiber uniformity. ** indicates that the correlation was significant at the *P* < 0.01 level

Correlation analysis of the five fiber quality traits showed that highly significant correlations existed among the five fiber quality traits (Fig. 2). Significant positive correlations were observed among the four traits FE, FS, FL and FU, ranging from 0.51 to 0.84, whereas FM was significantly negatively correlated with FS and FL. In addition, ANOVA revealed the effects of genotype (G), environment (E) and the interaction of genotype and environment (G × E) were significant (*P* < 0.001) on fiber traits (Additional file 1: Table S1). This result suggested that fiber quality traits are affected by both genotype and environment. The broad-sense heritability (*H*^*2*^) values of FE, FM, FS, FL and FU were 73.31%, 91.38%, 84.78%, 84.54% and 72.06%, respectively (Table 1), suggesting that fiber quality traits are mainly controlled by genetic effects.

### GWAS of fiber quality traits

**Fig. 3:**
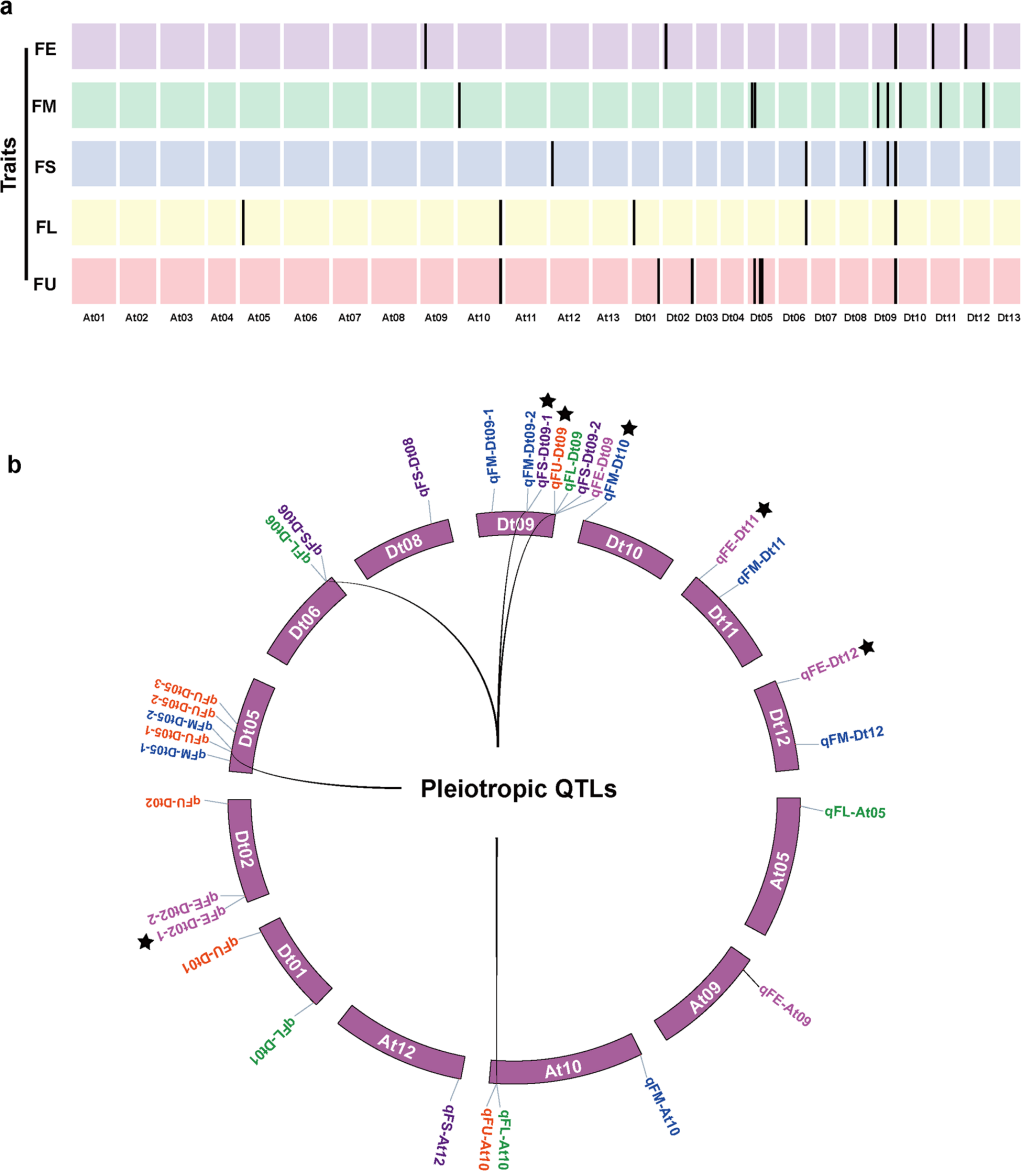
Distribution of significant SNPs and QTLs identified in this study. **a** Distribution of the trait-associated SNPs of five fiber traits. The colored rectangles represent cotton chromosomes, and the black vertical lines indicate the significantly associated SNPs. **b** Distribution of the trait-associated QTLs of five fiber traits. QTLs presented in different colors are associated with different cotton fiber quality traits. The black asterisks indicate the novel QTLs identified in the study, and the black curves in the circle indicate the pleiotropic QTLs

To identify genetic factors underlying fiber quality traits, we performed a GWAS of the five fiber quality traits, combining 10,660 high-quality SNP markers identified using the CottonSNP63K array and phenotypic data collected from multiple environments. In total, 42 SNPs significantly associated with the five fiber quality traits were detected using the BLUP values (Fig. 3a, Additional file 2: Figure S1 and Additional file 1: Table S2). They were scattered across 13 chromosomes, including At05, At09, At10, At12, Dt01, Dt02, Dt05, Dt06, Dt08, Dt09, Dt10, Dt11 and Dt12. Moreover, the 9 and 33 significant SNPs were located in the At and Dt subgenomes, respectively.

For FE, 10 significant SNPs distributed on chromosomes At09, Dt02, Dt09, Dt11 and Dt12 were identified. The phenotypic variation explained (PVE) by each SNP ranged from 3.00% to 4.41% (Additional file 1: Table S2). Among these SNPs, locus i52264Gb had a positive effect on FE, with the highest −log_10_(*P*) value (4.18), and i39571Gh had a negative effect on FE, with the lowest −log_10_(*P*) value (3.02).

For FM, 11 significant SNPs were detected on chromosomes At10, Dt05, Dt09, Dt10, Dt11 and Dt12, explaining 3.06%–3.98% of the phenotypic variation (Additional file 1: Table S2). Of these SNPs, locus i35898Gh had a negative effect on FM, with the highest −log_10_(*P*) value (3.73), and i09491Gh had a positive effect on FM, with the lowest −log_10_(*P*) value (3.00).

For FS, eight significant SNPs located on chromosomes At12, Dt06, Dt08 and Dt09 were found. They explained 3.33%–4.65% of the phenotypic variation (Additional file 1: Table S2). Locus i52359Gb had a negative effect on FS, with the highest −log_10_(*P*) value (4.03), while i30183Gh had a positive effect on FS, with the lowest −log_10_(*P*) value (3.04).

For FL, six significant SNPs were observed on chromosomes At05, At10, Dt01, Dt06 and Dt09. They explained 3.31%–4.16% of the phenotypic variation (Additional file 1: Table S2). Locus i52359Gb had a negative effect on FL, with the highest −log_10_(*P*) value (3.66), and i33845Gh had a positive effect on FL, with the lowest −log_10_(*P*) value (3.02).

For FU, 11 significant SNPs were identified on chromosomes At10, Dt01, Dt02, Dt05 and Dt09, contributing 2.64%–4.19% of the phenotypic variation (Additional file 1: Table S2). Locus i09849Gh had a negative effect on FU, with the highest −log_10_(*P*) value (4.42), and i65397Gm had a positive effect on FU, with the lowest −log_10_(*P*) value (3.00).

### Identification and pleiotropy of QTLs

According to the definition of QTL from a previous study [19, 22], 31 QTLs were identified in this study: six for FE, eight for FM, five for FS, five for FL and seven for FU (Fig. 3b). These QTLs were distributed on different chromosomes that included significantly associated SNPs. Among these QTLs, six (*qFE-Dt02–1*, *qFE-Dt11*, *qFE-Dt12*, *qFM-Dt10*, *qFS-Dt09–1* and *qFU-Dt09*) were newly identified, and the remaining QTLs overlapped with previously identified QTLs (Fig. 3b and Additional file 1: Table S3).

Gene linkage and pleiotropic effects are commonly observed between complex agronomic traits [23]. In our study, two significant SNPs (i52359Gb and i11316Gh) exhibited pleiotropy and were associated with four and two fiber traits, respectively (Additional file 1: Table S3). Meanwhile, QTLs containing these significant SNPs were anchored in the same genomic region. For example, there were four QTLs (*qFE-Dt09*, *qFS-Dt09–2*, *qFL-Dt09* and *qFU-Dt09*) on Dt09, which were associated with different fiber traits and simultaneously mapped to the genomic region of Dt09: 50.51–50.91 Mb. On Dt06, two QTLs (*qFS-Dt06* and *qFL-Dt06*) with the same significant SNP were located in the same genomic interval (59.8–60.2 Mb). Moreover, some QTLs overlapped with or were adjacent to other QTLs in terms of physical position, such as *qFL-At10*/*qFU-At10*, *qFM-Dt05–2*/*qFU-Dt05–1* and *qFM-Dt09–2*/*qFS-Dt09–1* (Fig. 3b). These results implied that these fiber traits might be controlled by a QTL network with multiple effects.

### Expression profile analysis of genes in the QTL regions

**Fig. 4:**
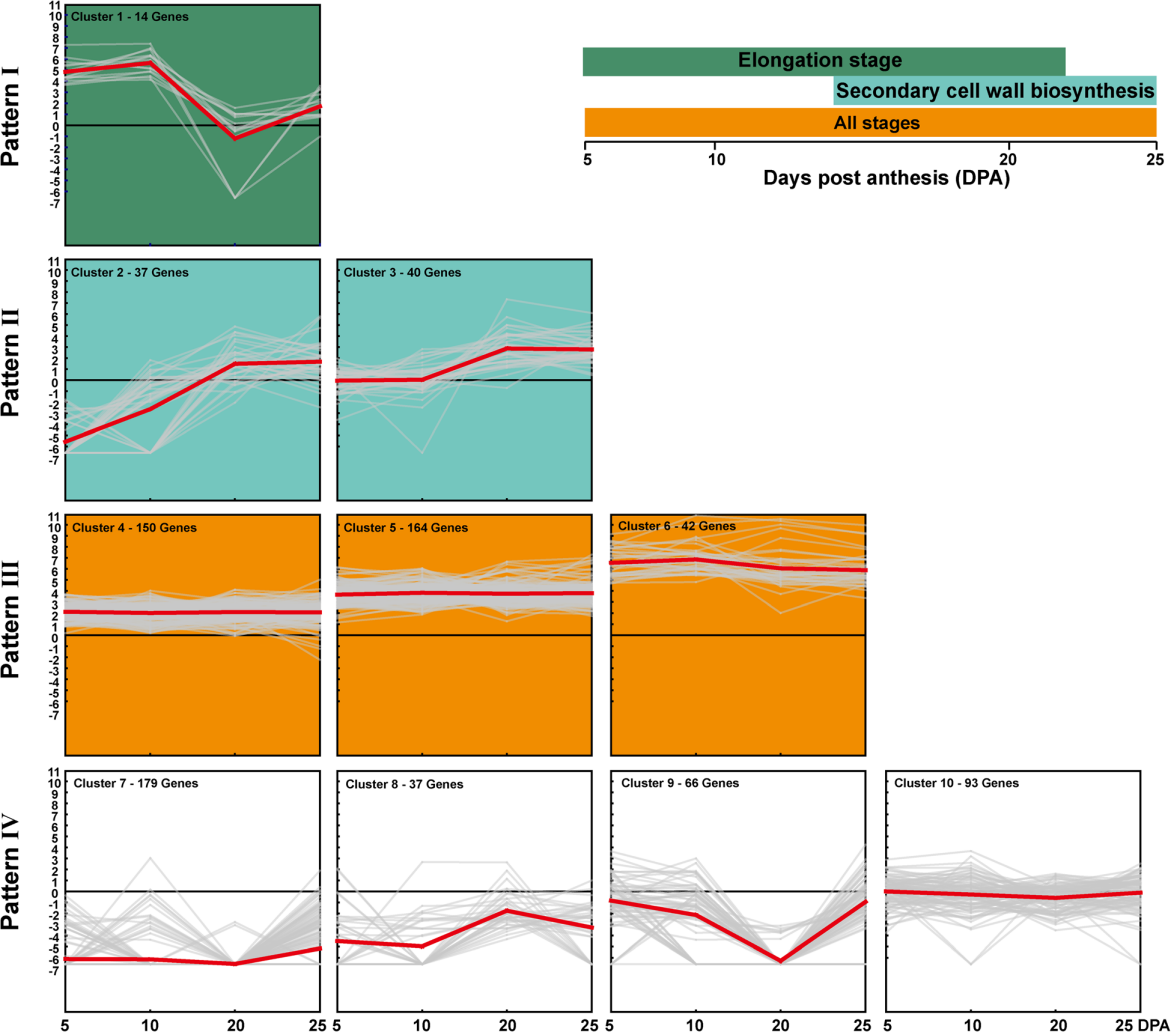
Expression profiles of these genes in the QTL regions. Cluster analysis was performed using RNA-seq data of 5, 10, 20 and 25 DPA during the fiber developmental stage. Different colors represent different expression patterns and different fiber developmental stages

Based on the *G. hirsutum* TM-1 reference genome sequence [10], 822 genes were detected in these QTL regions (Additional file 1: Table S4). These genes were unevenly distributed across the genome, with 154 and 668 genes in the At and Dt subgenomes, respectively (Additional file 3: Figure S2). Moreover, the number of genes differed among chromosomes. The largest number of genes was located on Dt05 (188 genes), while there were only 14 genes on At09. In addition, we investigated the expression patterns of these genes using transcriptome data on fiber development, which were obtained from the NCBI Sequence Read Archive (SRA) database. The results revealed that these genes could be divided into four patterns, I–IV (Fig. 4). Pattern I included 14 genes highly expressed at the elongation stage for about 5 to 10 days post anthesis (DPA). Pattern II, comprising two clusters (Clusters 2 and 3), contained 77 genes preferentially expressed at the secondary cell wall synthesis stage (20–25 DPA). Pattern III contained 356 genes that maintained high expression levels at all of the fiber developmental stages. Pattern IV included 375 genes with low expression levels at all fiber developmental stages. This finding suggested that these genes could be involved in fiber development and play important roles in different stages of fiber development.

### Analysis of pleiotropic SNPs associated with fiber quality

In plants, many traits are controlled by multiple genes, and some genes have pleiotropic effects on yield traits or other traits [20]. Gene pleiotropy means that a gene determines or influences the formation of multiple traits [24]. In this study, we identified single genomic regions on both chromosomes Dt09 and Dt06 that exhibited pleiotropic associations with more than one fiber quality trait.

**Fig. 5:**
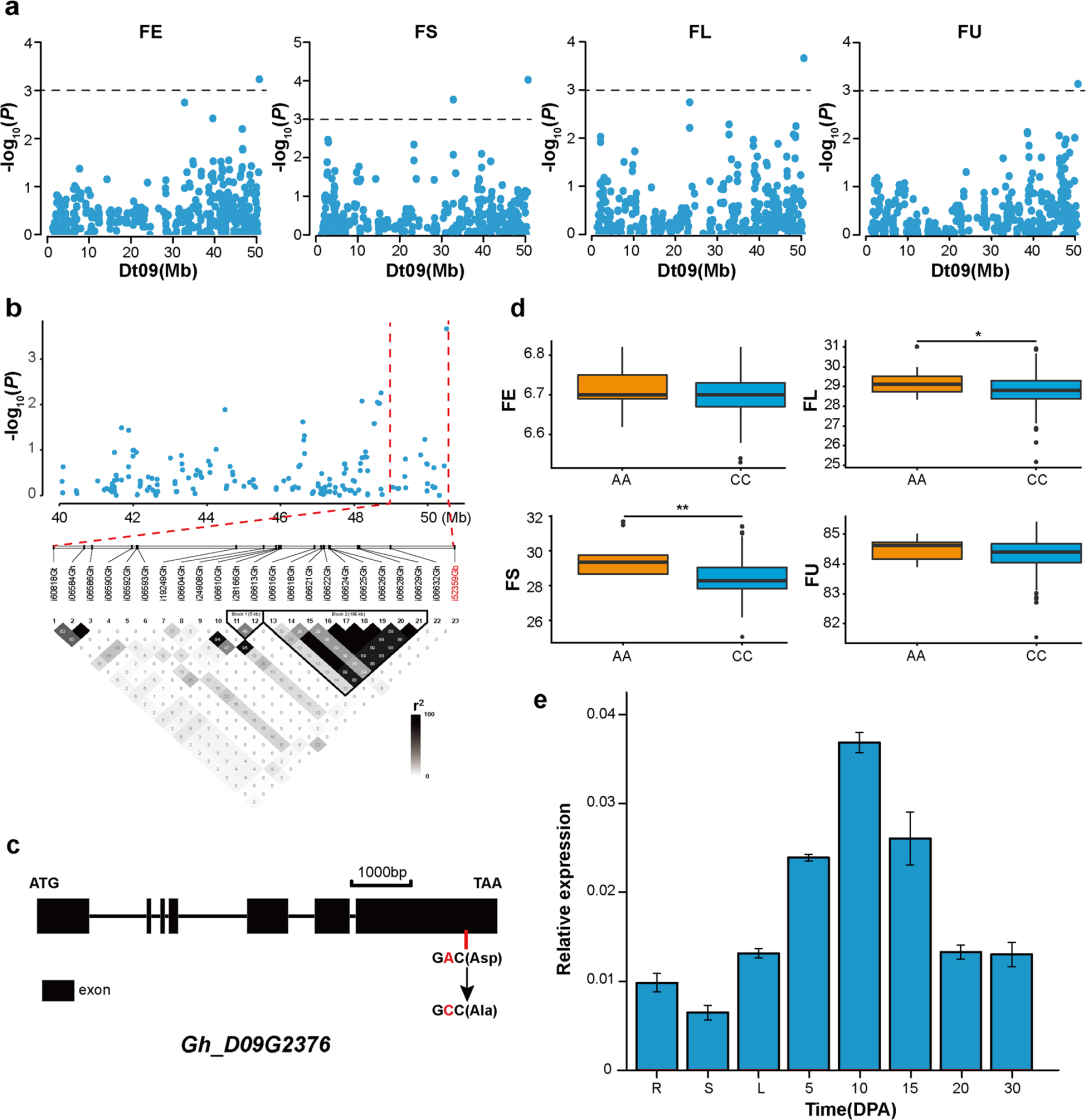
Identification of the cotton fiber quality trait-associated candidate gene *Gh_D09G2376* on Dt09. **a** Manhattan plots for FE, FS, FL and FU on Dt09. The dashed line indicates the significance threshold (*P* < 10^− 3^). **b** Local Manhattan plot and LD heatmap of the genomic region 49.0–50.7 Mb on Dt09. The red dashed line indicates the candidate region. **c** Gene structure of *Gh_D09G2376*. Black rectangles and black lines represent exons and introns, respectively. **d** Box plots of FE, FS, FL and FU based on the SNP allele i52359Gb. The significance levels of the differences were assessed using two-tailed t-tests. **e** RT-qPCR of *Gh_D09G2376*. *GhHis3* was used as a housekeeping gene. Error bars represent the standard deviations of three independent biological replicates

On Dt09, the association signal was located at 49.0–50.7 Mb (Fig. 5). In this candidate region, one SNP was significantly associated with four fiber quality traits (FE, FS, FL and FU) (Fig. 5a) and was located within the gene *Gh_D09G2376*, which encodes a Jumonji N/C and zinc finger domain-containing protein (Fig. 5c). The linkage disequilibrium (LD) block analysis showed that two blocks exist in this region, but the significantly associated SNP did not belong to any block (Fig. 5b). Moreover, i52359Gb is a nonsynonymous SNP that mutated from A to C at the 7215 bp position in the coding region of *Gh_D09G2376*, which resulted in an amino acid change from aspartic acid (Asp) to alanine (Ala) (Fig. 5c). Interestingly, the two genotypes generated by this SNP locus were associated with the phenotypic performances of these fiber traits. The accessions with the AA genotype showed higher FE, FS, FL and FU values than the accessions with the CC genotype. In particular, FS and FL were significantly different between these two genotypes (Fig. 5d). RT-qPCR analysis revealed that this gene was highly expressed at the fiber elongation stage (10 DPA) (Fig. 5e). Moreover, this gene was significantly higher expression at 10 DPA of Xinluzao30 (high-quality upland cotton cultivar) compared with Sukang191 (low-quality upland cotton cultivar) (Additional file 4: Figure S3a and b). In cotton, the function of the *Gh_D09G2376* gene is unknown. However, its orthologous gene in *Arabidopsis*, *JMJ12* (*At3g48430*) encodes a Jumonji N/C and zinc finger domain-containing protein and plays an important role in cell elongation (Additional file 5: Figure S4) [25, 26]. Therefore, we hypothesize that this is a pleiotropic gene controlling FE, FS, FL and FU.

**Fig. 6:**
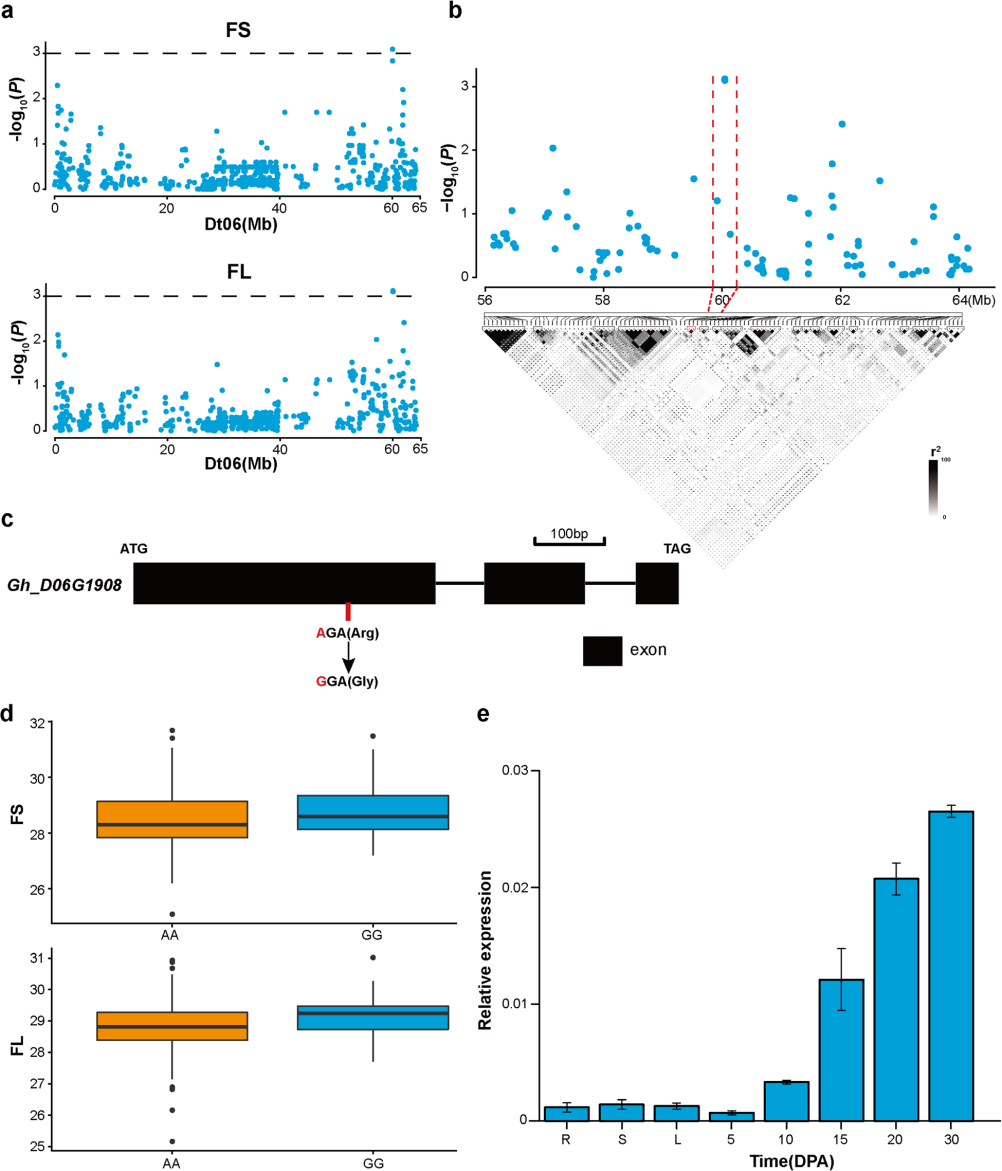
Identification of the cotton fiber quality trait-associated candidate gene *Gh_D06G1908* on Dt06. **a** Manhattan plots for FS and FL on Dt06. The dashed line indicates the significance threshold (*P* < 10^− 3^). **b** Local Manhattan plot and LD heatmap of the genomic region 56.1–64.2 Mb on Dt06. The red dashed line indicates the candidate region. **c** Gene structure of *Gh_D06G1908*. Black rectangles and black lines represent exons and introns, respectively. **d** Box plots of FS and FL based on the SNP allele i11316Gh. The significance levels of the differences were assessed using two-tailed t-tests. **e** RT-qPCR of *Gh_D06G1908*. *GhHis3* was used as a housekeeping gene. Error bars represent the standard deviations of three independent biological replicates

In addition, another association signal was associated with FS and FL within 59.8–60.2 Mb on Dt06 (Fig. 6). There were four SNPs in this candidate region, and two of them fell within an LD block (Fig. 6a and b). In this block, only the SNP i11316Gh was significantly associated with these traits and was located in the gene *Gh_D06G1908*. This SNP caused an amino acid change from arginine (Arg) to glycine (Gly) at the 310 bp position of this gene (Fig. 6c). And, based on the alleles of this SNP, these accessions were divided into two genotypes, AA and GG. The GG genotype, compared with the AA genotype, had greater FS and longer FL (Fig. 6d). Moreover, the gene was preferentially expressed at the fiber developmental stages, and its expression level increased during fiber development, peaking at 30 DPA (Fig. 6e). We also found that this gene showed higher expression in the high-quality upland cotton cultivar (Xinluzao30) than in the low-quality upland cotton cultivar (Sukang191) at 10, 15 and 20 DPA (Additional file 4: Figure S3a and c). In *Arabidopsis*, *CYSb* (*At3g12490*) is the homologue of *Gh_D06G1908*, and it encodes a protein with cysteine proteinase inhibitor activity (Additional file 6: Figure S5) The overexpression of *CYSb* can stimulate plant growth [27]. These results imply that *Gh_D06G1908* might participate in fiber developmental process.

## Discussion

### Phenotypic diversity and heritability of fiber quality traits

Genetic diversity, including genotypic and phenotypic diversity, plays a vital role in association mapping [28]. In the present study, the degree of genotypic diversity was similar to that measured in previous studies [19, 20], and it was suitable for a GWAS. Moreover, to increase the reliability of the phenotypic data and reduce environmental effects on the GWAS results, phenotypic data were collected from seven environments over 2 years, and BLUPs for the five fiber quality traits were estimated in this study. Phenotypic statistical results showed that all the traits exhibited phenotypic variation ranging from 0.61%–5.24% (Table 1), which was coincident with the results of previous studies [19, 20, 23].

Heritability is another main factor that influences the accuracy of association mapping [29]. Generally, *H*^*2*^ is used to judge the degree of stability of inherited traits, and an *H*^*2*^ value greater than 50% is considered high [30]. The fiber quality traits of upland cotton possess high heritability levels. For example, the *H*^*2*^ values of these traits reported by Nie et al. (2016), Huang et al. (2017) and Dong et al. (2018) were in the ranges of 86%–93%, 84%–92% and 69.54%–91.05%, respectively [5, 20, 23]. In our research, the *H*^*2*^ values of these fiber quality traits were also high, ranging from 72.06% (FU) to 91.38% (FM) (Table 1), which was consistent with the results of previous studies [5, 20, 23]. Moreover, ANOVA showed that the variances explained by genotype, environment and their interaction in these tested traits were all significant (Additional file 1: Table S1), suggesting that these traits were influenced by the environment. However, the genotype effect plays the dominant role. Thus, cotton fiber quality traits are predominately controlled by genetic factors and suitable for association mapping analysis.

### Pleiotropic SNPs and QTLs identified in the present study

Fiber quality traits are generally complex quantitative traits that are controlled by a complex network of multiple genes [31]. Some QTLs or genes simultaneously govern multiple fiber quality traits [13, 19]. In the present study, we identified 42 significant SNPs and 31 QTLs associated with these five fiber quality traits using the association mapping method (Additional file 1: Tables S3 and S4, Fig. 3). Interestingly, some of these significant SNPs or QTLs (within the same genomic interval) were associated with more than one trait. The significant SNP i52359Gb was simultaneously associated with FE, FS, FL and FU, and another SNP, i11316Gh, was concurrently associated with FS and FL. Moreover, *qFL-At10*, *qFM-Dt05–2* and *qFM-Dt09–2* overlapped with or were adjacent to *qFU-At10*, *qFU-Dt05–1*, and *qFS-Dt09–1*, respectively. Additionally, correlation analysis of these fiber quality traits showed that FE, FS, FL and FU were significantly positively correlated with each other, and a significant negative correlation was found for FM with both FL and FS. Thus, there were strong correlations between fiber quality traits, which was consistent with the results of previous reports [5, 15, 19]. In addition, correlations between cotton fiber quality traits are generally favorable [32, 33]. Thus, the genes involved in different fiber quality traits can be used to efficiently obtain the desired fiber quality through the improved breeding of cotton fiber.

### Comparison of our GWAS results with QTL or GWAS results from previous studies

In recent decades, large numbers of QTLs or GWAS signals associated with fiber quality traits in cotton have been identified using different genetic populations or different mapping methods [18, 34]. Among the 31 QTLs associated with these five fiber quality traits detected in the present study, 25 QTLs fell within or adjacent to QTL intervals or GWAS signals identified in previous studies (Additional file 1: Table S3). For FE, QTLs *qFE-At09*, *qFE-Dt02–2* and *qFE-Dt09* overlapped with DPL0679a [5], NAU3308 − NAU5467 [35] and *qFE23.1* [36, 37], respectively. For FM, six QTLs (*qFM-At10*, *qFM-Dt05–1*, *qFM-Dt05–2*, *qFM-Dt09–2*, *qFM-Dt11* and *qFM-Dt12*) corresponded to ten QTLs reported in previous studies, and some of these QTLs also mapped to regions adjacent to TM33781 [38], *qFM-Chr19–1* [39], NAU1004 [40], qFM23.2 [37] and TM79085 [38]. For FS, the QTLs *qFS-At12*, *qFS-Dt06* and *qFS-Dt08* were associated with *qFS-A12–1* [41], i38606Gh [19] and D08_54727428 [42], respectively. Moreover, *qFS-Dt09–2* was adjacent to *qFS23.2* [37]. For FL, the QTLs *qFL-At05*, *qFL-At10* and *qFL-Dt01* overlapped with *qFL-c10–1* [43], TM10319 and TM47849 [38], respectively, and QTLs *qFL-Dt06* and *qFL-Dt09* were close to GH185 [44] and TM72969 [38], respectively. For FU, the QTLs *qFU-At10*, *qFU-Dt02*, *qFU-Dt05–1*, *qFU-Dt05–2* and *qFU-Dt05–3* overlapped with *qFU-D5–2* [45], *qFU14.2* [36], HAU1384 [46], TM57244 and TM36632 [38], respectively, and one QTL (*qFU-Dt01*) was located close to the GWAS signal at D01:60914905 [13]. Thus, the GWAS results in the current study appear reliable, and these stably inherited QTLs/genes, which were detected simultaneously by different segregating populations having different genetic backgrounds or by different mapping methods, have great application potential in future breeding programs focused on improving cotton fiber quality. Moreover, we found that more QTLs were located in the Dt subgenome than in the At subgenome, which is consistent with the previous finding that the chromosomes in the Dt subgenome contained 25% more fiber quality QTLs than those in the At subgenome, and the data support the suspicion that the Dt subgenome plays a more important role in fiber-regulatory mechanisms [21, 30, 47, 48].

### Potential candidate fiber quality genes

To date, many candidate genes related to fiber developmental stages have been identified using different methods, such as *GhHD1* and *GhHOX3* by homologous gene cloning [49, 50], *GhMML3_A12* and *GhMML4_D12* by map-based cloning [51, 52] and *GhFL1* and *GhFL2* by GWAS [21]. Moreover, the development of cotton fiber is a complex and dynamic process, involving the initiation, elongation, secondary cell wall synthesis and maturation stages. Correspondingly, the regulatory mechanisms of fiber development are also complex, involving conserved transcription factors, phytohormones, epigenetic modifications and metabolic pathways [31].

In this study, 822 genes were identified in QTL regions (Additional file 1: Table S4). The gene expression profile analysis showed that many genes were preferentially expressed at specific or during all the fiber developmental stages. Among these genes, there were two candidate genes, *Gh_D09G2376* and *Gh_D06G1908* (Figs. 5 and 6). Both genes contain a significantly associated nonsynonymous SNP that causes an amino acid change. Based on the two alleles of these nonsynonymous SNPs, the accessions were classified into two types, carrying AA/CC and AA/GG alleles at i52359Gb and i11316Gh, respectively. Moreover, the gene expression analysis revealed that these two genes exhibited higher expression levels during fiber developmental stages than in other tissues, indicating that they may be involved in fiber development.

We focused on homologous annotation information on *Gh_D09G2376* and *Gh_D06G1908*. The *JMJ12* gene, a homologue of *Gh_D09G2376* in *Arabidopsis*, encodes a Jumonji N/C domain-containing protein. In *Arabidopsis*, *BES1* recruits *JMJ12* to active brassinosteroid (BR) responsive genes, and mutations in the *JMJ12* gene lead to impaired cell elongation [25, 26]. In cotton, BR is a critical regulator of fiber elongation [53], and *BES1* genes have influence on fiber development [54]. *Gh_D06G1908* is a homologue of *Arabidopsis CYSb* gene, which encodes a protein that inhibits the catalytic activity of proteinases. In *Arabidopsis*, *CYSb*-overexpression transgenic lines exhibit greater fresh weights than wild-type plants [27]. Therefore, we speculated that these two genes may underlie fiber development in cotton. Functional studies of these candidate genes are needed to further validate their roles in cotton fiber development.

## Conclusions

In this study, an association mapping method was used to explore the genetic architecture of fiber quality traits in upland cotton. A total of 42 SNPs and 31 QTLs significantly associated with these fiber quality traits were identified. In total, 822 genes were located in these QTLs. Furthermore, *Gh_D09G2376* and *Gh_D06G1908* were detected as candidate genes controlling fiber quality traits as assessed by RNA-seq and RT-qPCR analyses. In summary, the SNPs, QTLs and candidate genes identified in this study can be used in marker-assisted selection (MAS) of fiber quality traits and to increase our understanding of the molecular mechanisms responsible for fiber quality.

## Methods

### Plant materials and field experiments

A set of 276 upland cotton accessions were used for the GWAS and detailed information regarding these accessions has been published previously [22]. A randomized complete block design with two replicates was conducted and these accessions were planted in eight environments, including Anyang in Henan Province (in 2016 and 2017), Jingzhou in Hubei Province (in 2016 and 2017), Alaer in Xinjiang Province (in 2016 and 2017), Jiujiang in Jiangxi Province (in 2016) and Huanggang in Hubei Province (in 2017). In all environments, each plot was 6.0 m length with 0.8 m between rows, containing 20–25 plants. The field management followed local agronomic practices.

### Phenotypic data collection and statistical analysis

A total of 25 naturally open bolls were harvested from each plot by hand. After ginning, 10–15 g fiber from each sample was sent to the Cotton Fiber Quality Inspection and Testing Center of the Ministry of Agriculture, Anyang, China, and the fiber quality traits of each accession were measured by the HVI1000 automatic fiber testing system (http://www.uster.cn/en/instruments/fiber-testing-1/uster-hvi-3/), including FE (%), FM, FS (cN/tex), FL (upper-half mean FL, mm) and FU (%).

To reduce environment-related errors, the BLUPs of the fiber quality traits were calculated in R software using the lme4 package [55]. The BLUP values were used for subsequent association mapping. Statistical analyses, Pearson’s linear correlation coefficients between different fiber quality traits, and an analysis of variance (ANOVA) were also implemented in R software [56]. Moreover, the *H*^*2*^ values of each trait were calculated according to the previously reported method [22].

### Association mapping of fiber quality traits

SNP genotyping was performed using the CottonSNP63K array, and 10,660 high-quality SNPs were identified and employed in the association mapping. Population structure, relative kinship and LD analyses of this population have been reported previously [22]. Here, a GWAS was conducted using the Genome Association and Prediction Integrated Tool (GAPIT) with a mixed linear model (MLM) (PCA + K) [57]. To obtain more significantly associated SNP, a threshold of *P* = 1.0 × 10^− 3^ was chosen. Manhattan plots were drawn using qqman package in R software [58]. The LD heatmaps near peak SNPs were produced using Haploview 4.2 software [59].

### Identification of QTLs and gene expression analysis

In accordance with a previously reported method [19, 22], we selected 200-kb regions upstream and downstream of the significant trait-associated SNPs as QTLs based on the *G. hirsutum* TM-1 reference genome sequence [10]. The co-location analysis of our GWAS results and previously reported results was implemented by the following steps described in previous studies [60]: (1) the previously reported QTLs and GWAS signals were obtained from the cotton QTL database (CottonQTLdb, http://www2.cottonqtldb.org/) [6], and the primer sequences of these markers were selected from the Cottongen database (https://www.cottongen.org/) [61]; (2) the physical positions of these QTLs were obtained using electronic PCR (e-PCR) based on the *G. hirsutum* TM-1 reference genome sequence [10]; and (3) these previous QTLs were compared with the QTLs identified in this study on a physical map.

The genes in the QTL regions were mined from the gene annotations in the *G. hirsutum* TM-1 reference genome [10]. To investigate the expression profiles of these genes, RNA-seq data of *G. hirsutum* TM-1 tissues were obtained from the NCBI Sequence Read Archive (SRA) database under accession PRJNA248163 [10]. And, RNA-seq data were processed using TopHat and Cufflinks software [62], and normalized fragments per kilobase per million mapped read (FPKM) values were used to indicate the gene expression levels.

### RNA extraction and RT-qPCR verification

Root, stem, leaf and fiber samples at 5, 10, 15, 20 and 30 DPA obtained from *G. hirsutum* TM-1, and fiber samples at 10, 15 and 20 DPA obtained from Xinlumian30 (high-quality upland cotton cultivar) and Sukang191 (low-quality upland cotton cultivar) were used for total RNA isolation. Total RNA extraction, cDNA synthesis and RT-qPCR were performed following the previously study [22]. Three independent biological replicates were performed for each sample. *GhHis3* was chosen as the internal reference gene. Relative gene expression values were calculated using the comparative Ct method [63]. All primers used in this study are listed in Additional file 1: Table S5.

## Supplementary information

**Additional file 1: Table S1.** Analysis of variance (ANOVA) of the cotton fiber quality traits. **Table S2.** Significant SNPs detected for cotton fiber quality traits by a GWAS. **Table S3.** Comparisons of the QTLs identified in this study with those identified in previous studies. **Table S4.** Annotation information of these genes in the QTL regions. **Table S5.** Primer sequences for RT-qPCR in this study.

**Additional file 2: Figure S1.** Manhattan plots and quantile-quantile plots for FE, FM, FS, FL and FU. The dashed horizontal line indicates the significance threshold (*P* < 10^− 3^).

**Additional file 3: Figure S2.** Distribution of genes in these QTL regions.

**Additional file 4: Figure S3.** Comparison of expression levels of *Gh_D09G2376* and *Gh_D06G1908* between Xinlumian30 and Sukang191. **a** Box plots for fiber quality traits of Xinlumian30 and Sukang191. **b** Expression of *Gh_D09G2376* in Xinlumian30 and Sukang191 by RT-qPCR. **c** Expression of *Gh_D06G1908* in Xinlumian30 and Sukang191 by RT-qPCR. *GhHis3* was used as a housekeeping gene. Error bars represent the standard deviations of three independent biological replicates. ** indicates the significance level at 0.01 by using two-tailed t-test method.

**Additional file 5: Figure S4.** Homology analysis of *Gh_D09G2376***. a** Phylogenetic tree of *Gh_D09G2376* and *Arabidopsis JMJ* gene family. **b** Protein structure of *Gh_D09G2376* and *At3g48430/JMJ12*.

**Additional file 6: Figure S5.** Homology analysis of *Gh_D06G1908***. a** Phylogenetic tree of *Gh_D06G1908* and *Arabidopsis CYS* gene family. **b** Protein structure of *Gh_D06G1908* and *At3g12490/CYSb*.

## Data Availability

The raw RNA-seq data are available from NCBI Sequence Read Archive (SRA) database under accession PRJNA248163. All data generated and analyzed during this study are included in this published article and its supplementary information files. The datasets generated and analyzed during the current study are available from the corresponding author on reasonable requests.

## Abbreviations

FE: Fiber elongation
FM: Fiber micronaire
FS: Fiber strength
FL: Fiber length
FU: Fiber uniformity
GWAS: Genome-wide association study
QTLs: Quantitative trait loci
SNP: Single nucleotide polymorphism
PVE: Phenotypic variation explained
BLUPs: Best linear unbiased predictions
*H*^*2*^: Broad-sense heritability
DPA: Days post anthesis
LD: Linkage disequilibrium
