# Supplementary figures and images for: Genome-wide association study reveals the genetic basis of fiber quality traits in upland cotton (Gossypium hirsutum L.)
Source: BMC Plant Biol. 2020 Aug 27;20:395. doi: 10.1186/s12870-020-02611-0 (PMC7450593; doi:10.1186/s12870-020-02611-0)

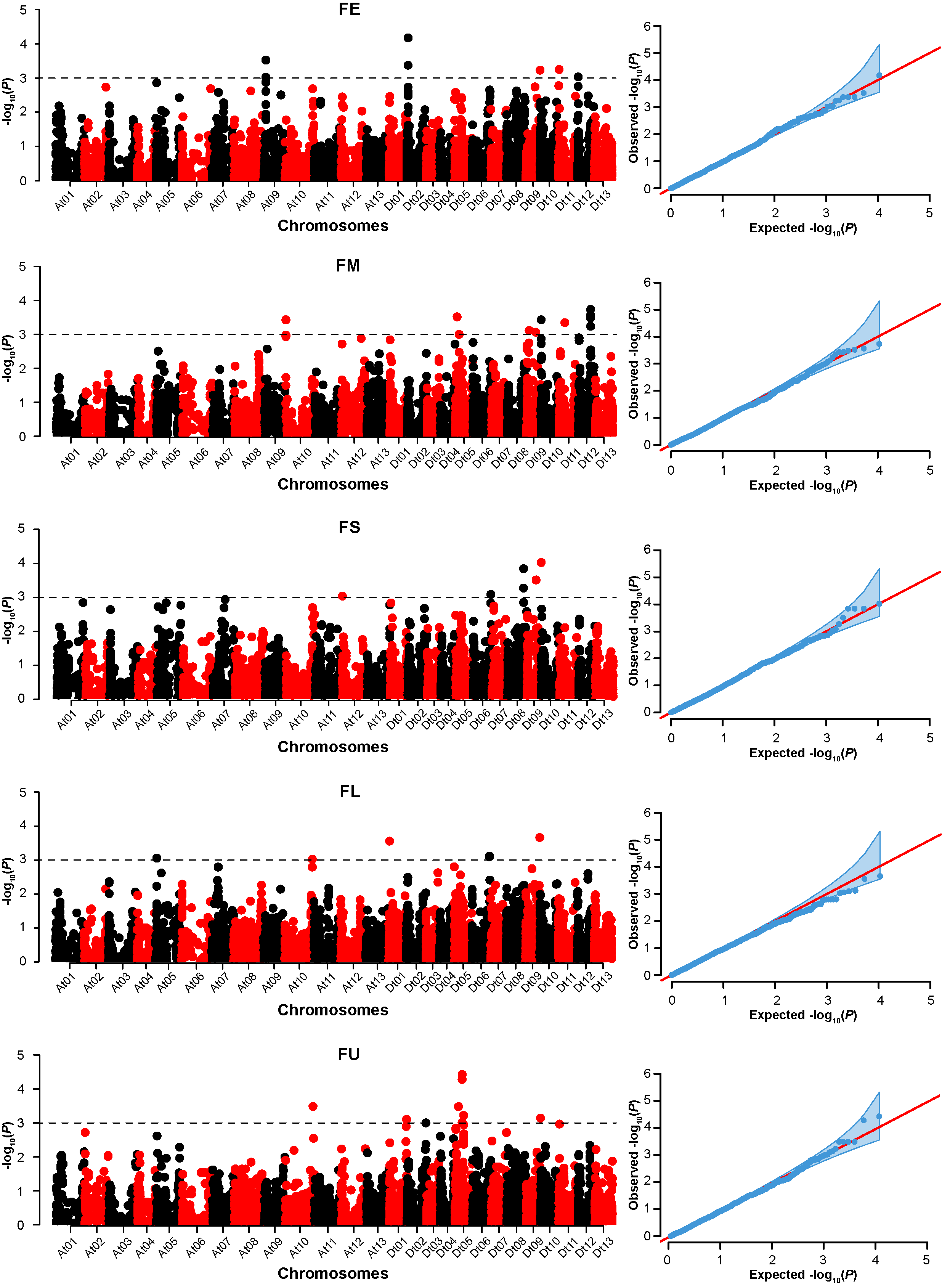

Supplement: Supplementary file 2 — Additional file 2: Figure S1. Manhattan plots and quantile-quantile plots for FE, FM, FS, FL and FU. The dashed horizontal line indicates the significance threshold (P < 10− 3). [file 12870_2020_2611_MOESM2_ESM.tif]

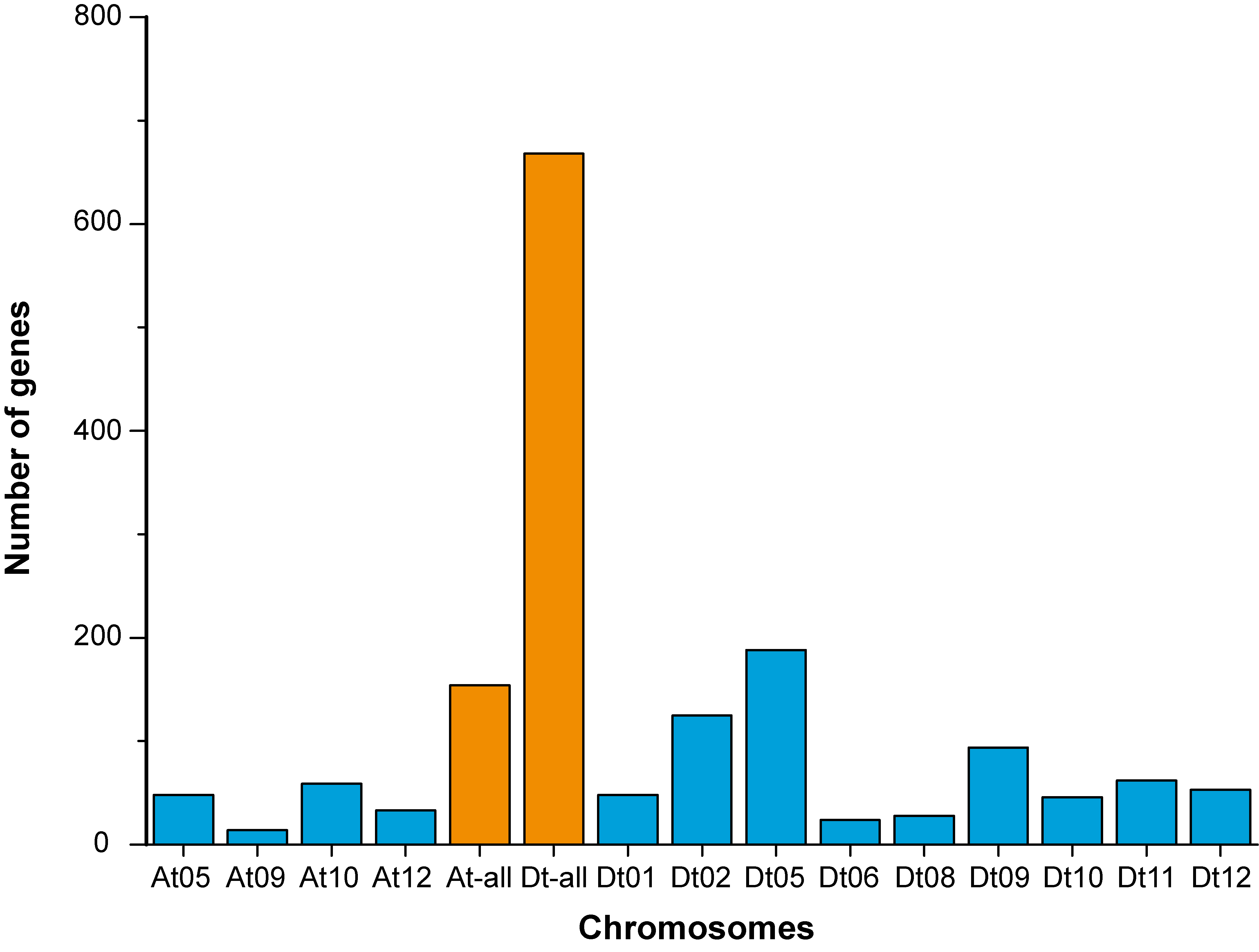

Supplement: Supplementary file 3 — Additional file 3: Figure S2. Distribution of genes in these QTL regions. [file 12870_2020_2611_MOESM3_ESM.tif]

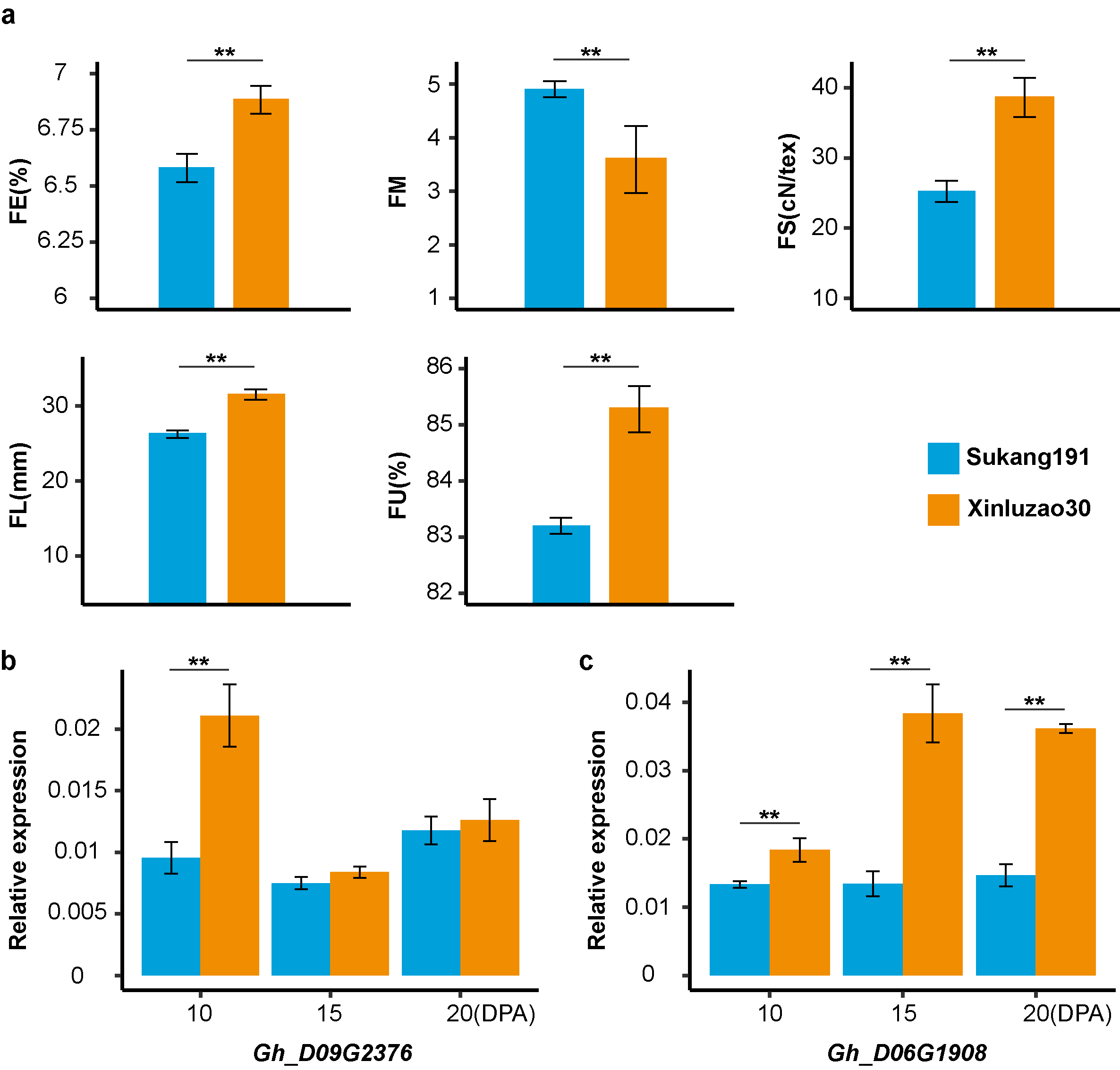

Supplement: Supplementary file 4 — Additional file 4: Figure S3. Comparison of expression levels of Gh_D09G2376 and Gh_D06G1908 between Xinlumian30 and Sukang191. a Box plots for fiber quality traits of Xinlumian30 and Sukang191. b Expression of Gh_D09G2376 in Xinlumian30 and Sukang191 by RT-qPCR. c Expression of Gh_D06G1908 in Xinlumian30 and Sukang191 by RT-qPCR. GhHis3 was used as a housekeeping gene. Error bars represent the standard deviations of three independent biological replicates. ** indicates the significance level at 0.01 by using two-tailed t-test method. [file 12870_2020_2611_MOESM4_ESM.tif]

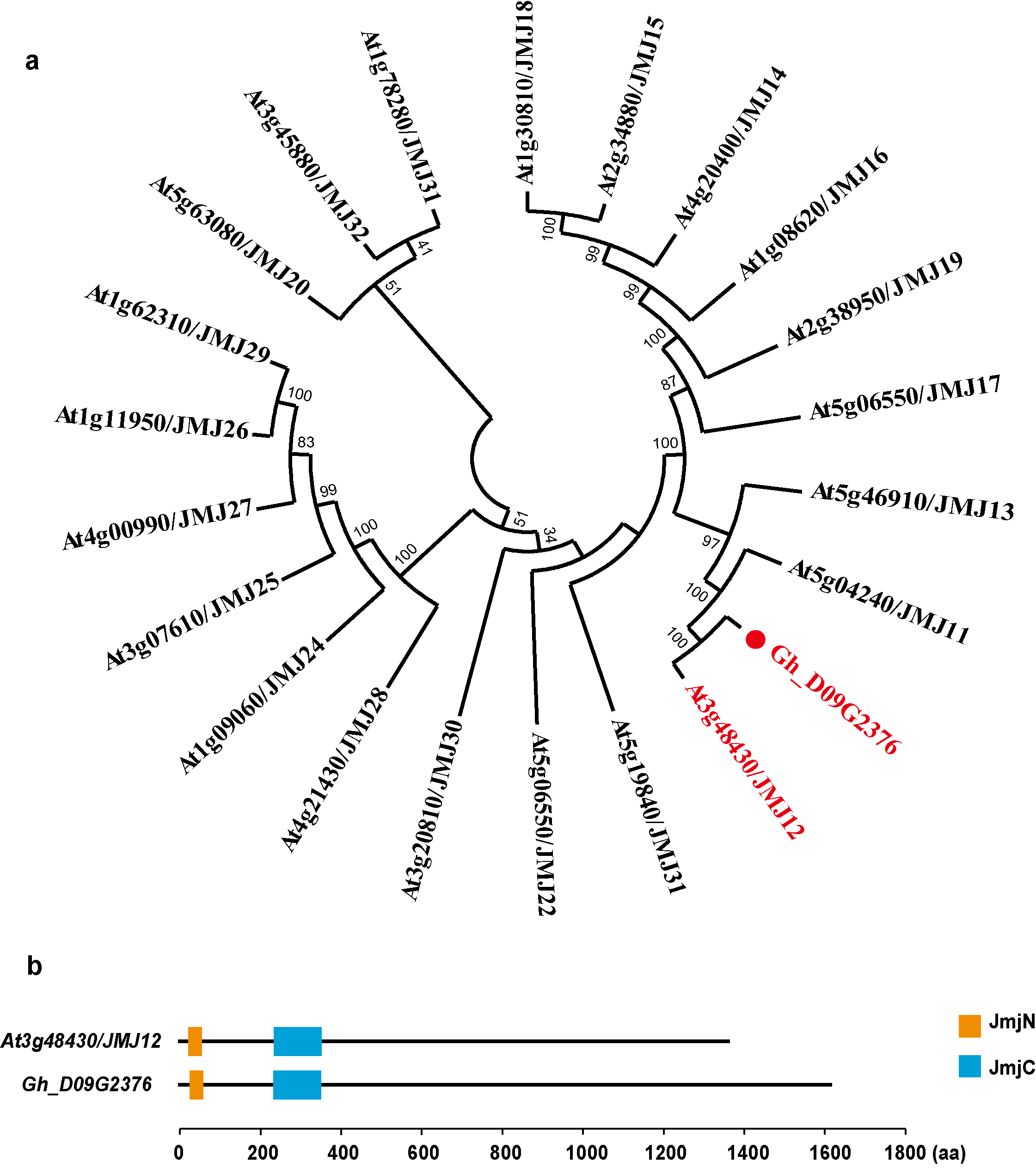

Supplement: Supplementary file 5 — Additional file 5: Figure S4. Homology analysis of Gh_D09G2376. a Phylogenetic tree of Gh_D09G2376 and Arabidopsis JMJ gene family. b Protein structure of Gh_D09G2376 and At3g48430/JMJ12. [file 12870_2020_2611_MOESM5_ESM.tif]

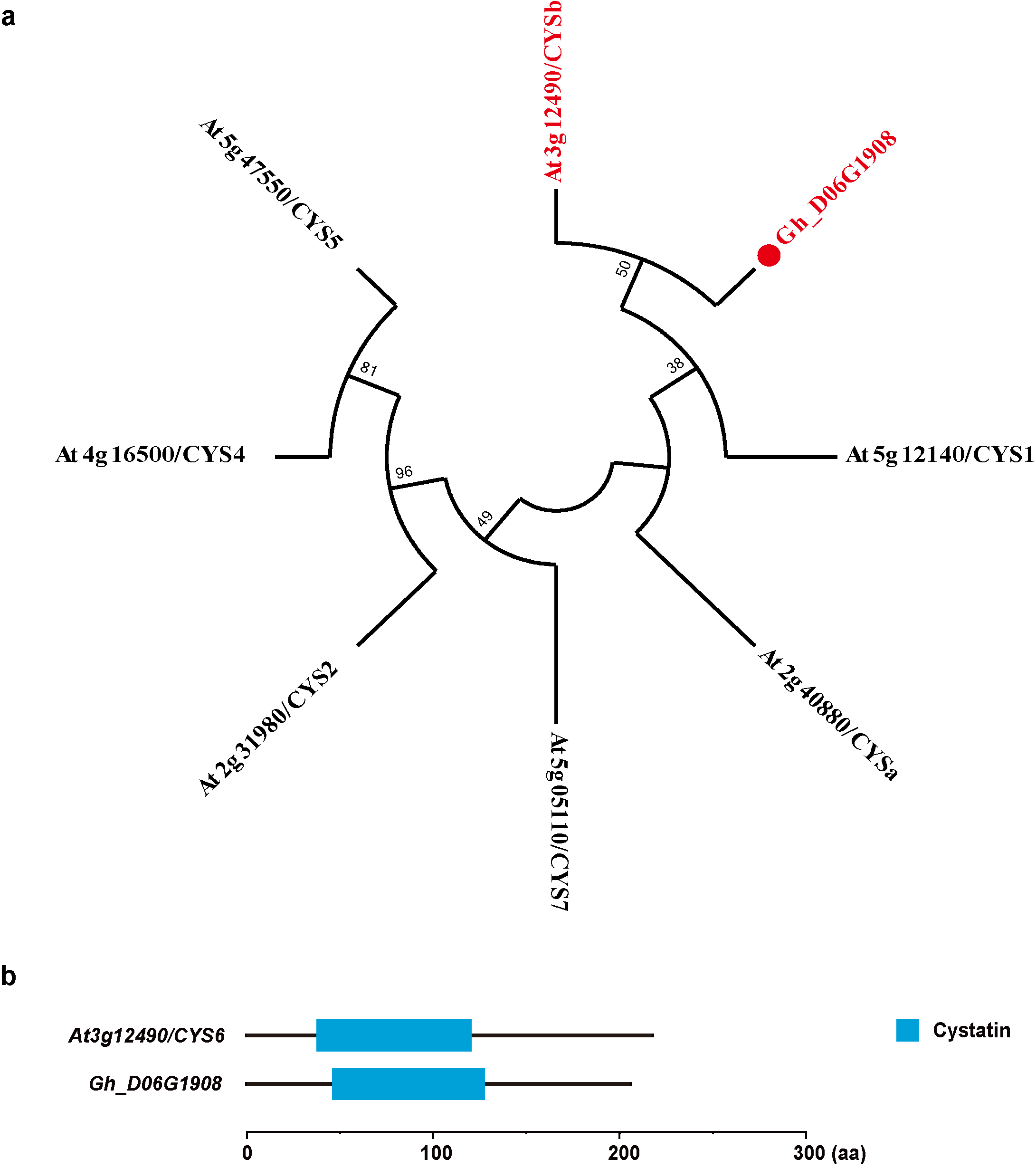

Supplement: Supplementary file 6 — Additional file 6: Figure S5. Homology analysis of Gh_D06G1908. a Phylogenetic tree of Gh_D06G1908 and Arabidopsis CYS gene family. b Protein structure of Gh_D06G1908 and At3g12490/CYSb. [file 12870_2020_2611_MOESM6_ESM.tif]
